# A High-Performance Genetic Algorithm: Using Traveling Salesman Problem as a Case

**DOI:** 10.1155/2014/178621

**Published:** 2014-05-05

**Authors:** Chun-Wei Tsai, Shih-Pang Tseng, Ming-Chao Chiang, Chu-Sing Yang, Tzung-Pei Hong

**Affiliations:** ^1^Department of Computer Science and Engineering, National Sun Yat-sen University, Kaohsiung 80424, Taiwan; ^2^Department of Applied Informatics and Multimedia, Chia Nan University of Pharmacy & Science, Tainan 71710, Taiwan; ^3^Department of Electrical Engineering, National Cheng Kung University, Tainan 70101, Taiwan; ^4^Department of Computer Science and Information Engineering, National University of Kaohsiung, Kaohsiung 81148, Taiwan

## Abstract

This paper presents a simple but efficient algorithm for reducing the computation time of genetic algorithm (GA) and its variants. The proposed algorithm is motivated by the observation that genes common to all the individuals of a GA have a high probability of surviving the evolution and ending up being part of the final solution; as such, they can be saved away to eliminate the redundant computations at the later generations of a GA. To evaluate the performance of the proposed algorithm, we use it not only to solve the traveling salesman problem but also to provide an extensive analysis on the impact it may have on the quality of the end result. Our experimental results indicate that the proposed algorithm can significantly reduce the computation time of GA and GA-based algorithms while limiting the degradation of the quality of the end result to a very small percentage compared to traditional GA.

## 1. Introduction

In the area of combinatorial optimization research [1], the traveling salesman problem (TSP) [2] has been widely used as a yardstick by which the performance of a new algorithm is evaluated, for TSP is NP-complete [3]. As such, any efficient solution to the TSP can be applied to solve many real world problems, such as transportation control [4], network management [5], and scheduling [6]. Assuming that *d*(*c*_*i*_, *c*_*j*_) represents the distance between each pair of cities *c*_*i*_ and *c*_*j*_, the TSP asks for a solution—that is, a permutation 〈*c*_*π*(1)_, *c*_*π*(2)_,…, *c*_*π*(*n*)_〉 of the given *n* cities—that minimizes

(1) $$\begin{array}{c} D=\left(\overset{n-1}{\underset{i=1}{\sum }}d\left(c_{\pi (i)},c_{\pi (i+1)}\right)\right)+d\left(c_{\pi (n)},c_{\pi (1)}\right). \end{array}$$

In short, (1) gives the distance *D* of the tour that starts at city *c*_*π*(1)_, visits each city in sequence, and then returns directly to *c*_*π*(1)_ from the last city *c*_*π*(*n*)_. Since the brute force method is impractical for the TSP except when the number of cities is small, the research direction for the TSP has been using heuristic search methods [7–9] to find a near-optimal solution.

Since the 1950s, heuristic algorithms have been developed for finding an approximate solution to the TSP and other complex optimization problems in a reasonable time [10]. Among the most widely used heuristic algorithms are evolutionary algorithms, swarm intelligence, and many others [11–16]. These algorithms eventually have a strong impact on modern computer science research because they help researchers solve problems in a variety of domains for which solutions in their full generality cannot be found in a reasonable time, even with the world's fastest computers. For reasons such as being an inherently parallel algorithm, being global search heuristics, and being easy to implement, GA [17, 18] has nowadays become one of the most popular heuristic algorithms. Moreover, Holland's schema theorem [17], which says that “short, low-order, above-average schemata receive exponentially increasing trials in subsequent generations of a GA” and Goldberg's building block hypothesis [18], which says that “a GA seeks near-optimal performance through the juxtaposition of short, low-order, high-performance schemata, called the building blocks” tell us that good subsolutions (or partial solutions) of a GA have a high probability of surviving the evolution and ending up being part of the final solution. This is further confirmed by Glover's proximate optimality principle (POP) [19], which says that “good solutions at one level are likely to be found close to good solutions at an adjacent level,” or good solutions have similar structures. A crucial observation above is that good subsolutions of a GA (or simply GA) (since no confusion is possible, we will use GA to represent simple or traditional GA throughout this paper) will become more and more similar to each other during its evolution process. This, in turn, implies that many of the computations of good subsolutions at the later generations of a GA are essentially redundant. The question is how do we eliminate these redundant computations at the early generations of a GA so that the computation time can be significantly reduced while at the same time retaining or enhancing the quality of the end result.

**Figure 1:**
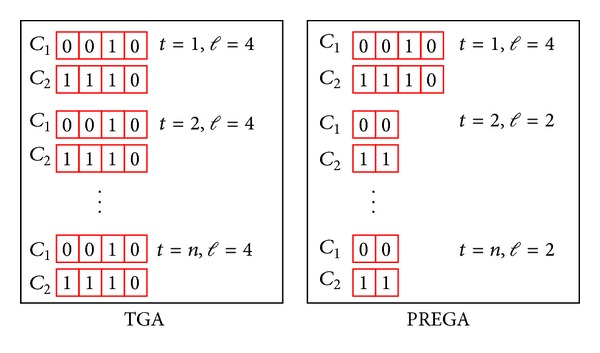
A simple example illustrating the difference between GA and PREGA. Note that genes common to chromosomes *C*_1_ and *C*_2_ are saved away by PREGA at generation *t* = 1 but not by TGA.

To make the idea more concrete, a simple example is given in Figure 1 to demonstrate how it works. As Figure 1 shows, let us suppose that there are two chromosomes, *C*_1_ and *C*_2_, each of which is composed of *l* genes. Let us further suppose that *l* = 4, and each gene can take only two possible values, namely, 0 and 1. Now let us assume, at a certain point in the evolution process, that the value of *C*_1_ is 0-0-1-0, and the value of *C*_2_ is 1-1-1-0 where the hyphen is used to separate the genes. Then what would be the values of *C*_1_ and *C*_2_ in the later generations? There are two answers to this question, depending on how the mutation operator is treated. The first answer is that if we use one point crossover and disregard the mutation operator altogether, then we are guaranteed that the values of the third and fourth genes of *C*_1_ and *C*_2_ will remain intact in the evolution process of a GA and will thus show up as part of the final solution. In other words, if the third and fourth genes of *C*_1_ and *C*_2_ (i.e., genes common to *C*_1_ and *C*_2_) are saved away, the number of genes will be cut into half and the computation time required by the crossover and mutation operators and the evaluation of the fitness function will be reduced. The second answer is that if we take into account the mutation operator, then the values of the third and fourth genes of *C*_1_ and *C*_2_ would have a small chance of not being 1-0. The probability for the values of the third and fourth genes of *C*_1_ and *C*_2_ being changed is, however, very small because only the mutation operator is allowed to change their values, and for GA, the mutation rate has almost always been set to a very small value, say, 1 or 2 percent.

The remainder of the paper is organized as follows.  Section 2 gives a brief introduction to the genetic algorithm and the approaches taken to enhance its performance.  Section 3 provides a detailed description of the proposed algorithm and a simple example to demonstrate how the proposed algorithm works. Performance evaluation of the proposed algorithm is presented in Section 4. Analysis of the proposed algorithm is given in Section 5. Conclusion is drawn in Section 6.

## 2. Related Work

As a particular class of evolutionary algorithms, it is well known that GA is a search technique aimed at finding true or approximate solutions to optimization problems. The operations used to emulate the evolution process of a GA are* selection*,* crossover*, and* mutation*. The simple or traditional GA [18] can be outlined as given in Algorithm 1. The selection operator takes the responsibility of guiding the search of GA toward the high quality or even optimal solution. The crossover operator plays the role of exchanging the information between the individuals in the population while the mutation operator is used to avoid GA from falling into local optima.

Researches on genetic algorithms focus not only on improving the quality of the end result but also on reducing the computation time of GA. Among them are parallel genetic algorithm, hybrid genetic algorithm, and radical modification of the evolutionary procedure or the design of GA.

1. Parallel genetic algorithm (PGA) [20, 21] is a very important technique for reducing the computation time of large problems, such as TSP [22]. The three distribution models [23] that have been proposed are master-slave model, fine-grained model (cellular model), and coarse-grained model (island model). However, in [24, 25], the authors indicate that the migration rate and strategy of the island model may affect its performance.
2. Hybrid genetic algorithm (HGA) [26] refers to the process of combining GA with other effective approaches for finding a better solution in terms of either the quality or the computation time. In general, the design of HGA may either integrate other heuristic algorithms [27] or combine local search methods [28] with GA. For instance, for an HGA that is a combination of GA and a local search method, GA is responsible for finding the global minima or pointing out the particular direction that may lead to a better solution while the local search method is used to find the local minima. For this reason, HGA will enhance the quality of the end result. In the research [29] on fast HGA (FHGA), Misevicius [29] points out that the design of FHGA should satisfy the following principles: (1) FHGA should arrive at the solutions quickly; (2) the populations should be compact to save the computation time; and (3) the diversity of the populations has to be maintained to avoid falling into the local minimum at early generations in the evolution process.
3. Another way to reduce the computation time is to radically change the evolutionary procedure or the design of GA. Michalski [30] presents a non-Darwinian-type evolution called learnable evolution model (LEM) that divides the whole population into two groups: high-performance group (H-group) and low-performance group (L-group). LEM first finds descriptions about why the H-group can obtain a better result and why the L-group may degrade the quality of the end result. Then, it uses the descriptions to generate chromosomes to replace those in L-group. Michalski also points out that LEM can speed up the number of evolutionary steps by a factor of two or more. Yet, when this kind of fast convergence methods [30, 31] of GA is used, it should be very careful about the convergence speed, or it may face the* premature convergence* problem. One possible solution to this problem is to use the fitness sharing [32] to avoid the diversity of the population being cut down too early.

The improvements that the abovementioned methods can achieve are limited intrinsically by the operators of GA. For example, the crossover and other genetic operators may disrupt the high quality subsolutions (building blocks, or BBs for short) that are found in the previous generations [33]. As a result, the convergence time of GA may increase [34]. Over the past two decades or so, various competent genetic algorithms (competent GAs) [33, 35–37] have been developed to tackle the linkage and scalability problem of GA. They can be broadly divided into two classes [36]. Also referred to as the perturbation technology, the first class is based on evolving the representation of solutions or adapting recombination operators among individual solutions. Among this class are the messy genetic algorithm, fast messy genetic algorithm (fmGA) [33, 38], and ordering messy GA (OmeGA) [36]. The fmGA differs from simple GA in several aspects. (1) Each gene of the fmGA is represented by its value and locus. (2) The fmGA uses variable-length chromosomes to represent the population. (3) The fmGA attempts to find the building blocks by repeatedly performing selection of solutions and random deletion of genes [36]. (4) A so-called* competitive template* is required to fill up the missing genes of underspecified messy chromosomes so that the fitness values can be evaluated.

## 3. The Proposed Algorithm

In this section, we present a simple but efficient technique for eliminating the redundant computations of GA and GA-based algorithms based on the notion of pattern reduction.  Algorithm 2 gives an outline of the pattern reduction enhanced genetic algorithm (PREGA). As Algorithm 2 shows, PREGA is built on the framework of GA; thus, it can be considered as an enhancement of GA with two operators—the common genes detection (CGD) and common genes compression (CGC) operators. If we disregard steps 3 and 4, PREGA given in Algorithm 2 will fall back into GA, as shown in Algorithm 1. The underlying idea of PREGA is to detect and compress genes common to all the chromosomes at the early generations of a GA to eliminate the redundant computations at the later iterations in the evolution process. In what follows, we will give a detailed description of the proposed algorithm.

### 3.1. Common Genes Detection (CGD)

The common genes detection operator of PREGA is responsible for detecting genes that are common to all the individuals in the population and thus are unlikely to be changed at later generations of the GA. Nevertheless, for different problems, the representation of chromosomes may have to be modified or even redesigned. From a different point of view, the example given in Figure 1 can be considered as a special case in terms of the fact that all the genes encode only two possible values 0 and 1 and all the genes are uncorrelated. In some other situations, such as traveling salesman problem, however, the solution of each gene will certainly affect the other genes, and genes on the same position of all the chromosomes do not necessarily represent identical subsolutions. For the TSP, each chromosome can be used to encode a different tour, that is, a different permutation 〈*c*_*π*(1)_, *c*_*π*(2)_,…, *c*_*π*(*n*)_〉 of the given cities. In other words, for all the chromosomes, *i* ≠ *j*—for all *i* and *j*, 1 ≤ *i* ≠ *j* ≤ *m*—implies *C*_*i*_ ≠ *C*_*j*_. Alternatively, each chromosome can be used to encode edges (corresponding to roads connecting pairs of cities) connecting pairs of cities of a tour.

In this paper, we use binary encoding for finding edges common to all the chromosomes. First, let us suppose that *E*(*i*, *j*) (here, we are assuming that node *i*, for all *i*, 1 ≤ *i* ≤ *m*, in the graph *G* representing the TSP, is labeled by city *c*_*i*_. Thus, we will use *i* and *c*_*i*_ interchangeably) is the edge connecting the pair of cities *c*_*i*_ and *c*_*j*_. Without loss of generality, let us further suppose that *i* < *j*. Otherwise, we can swap *c*_*i*_ and *c*_*j*_ or *i* and *j*, since insofar as this paper is concerned, only the symmetric TSP is considered. Then, all the *N* = *n*(*n* − 1)/2 edges *E*(*i*, *j*), 1 ≤ *i* < *j* ≤ *n*, can be assigned unique numbers in the range of 0 to *N* − 1, which can be computed as (2*n* − *i*)(*i* − 1)/2 + (*j* − *i* − 1) (it follows from (∑_*k*=*n*−*i*+1_^*n*−1^ *k*) + (*j* − *i* − 1) by simple algebraic manipulation).

To make the idea more concrete, (2) gives an example to show how all the 7(7 − 1)/2 = 21 edges *E*(*i*, *j*), 1 ≤ *i* < *j* ≤ 7, are assigned unique numbers in the range of 0 to 20.

(2) j 1234567 i1234567(−−−−−−−0−−−−−−16−−−−−2711−−−−381215−−−49131618−−51014171920−).

As the example shows, *E*(1,2) is assigned the number 0, *E*(1,3) the number 1, *E*(1,4) the number 2, and so on all the way up until *E*(6,7) is assigned the number 20. In other words, all the *n*(*n* − 1)/2 edges can be mapped to a one-dimensional array with exactly *n*(*n* − 1)/2 elements. This would save a little bit more than half of the space or more precisely *n*(*n* + 1)/2 entries. Now, to find edges common to all the chromosomes, we apply the common genes detection algorithm given in Algorithm 3.

Obviously, as Algorithm 3 shows, steps 1 and 3 take *O*(*n* ^2^) time, and step 2 takes *O*(*mn*) = *O*(*n*) time assuming that *m* is a constant. Thus, both the time and space complexities of the CGD algorithm are *O*(*n* ^2^), as claimed. It is worth mentioning that the CGD algorithm described in Algorithm 3 can be made even more efficient if we keep track of in a stack or an array (of size no more than *n*) the edges common to all the chromosomes in step 2 when the* last* chromosome is being scanned; then step 3 can be eliminated altogether. If we go one step further, eventually, the CGD algorithm can be made much more efficient and scalable than as outlined in Algorithm 3 by using a more complicated data structure such as balanced trees (the basic operations of which—such as member, insert, and delete—take *O*(log⁡*n*) time where *n* is the number of nodes in the tree). Again, assuming that *m* is a constant and that a balanced tree is used, the time complexity of the CGD algorithm can be cut from *O*(*n* ^2^) down to *O*(*mn*log⁡*mn*) = *O*(*n*log⁡*n*) and the space complexity from *O*(*n* ^2^) down to *O*(*mn*) = *O*(*n*), as claimed. As the number of generations increases, the number of cities *n* will be quickly decreased. This implies that the CGD operator is in general much faster than specified by the above bounds, which will in turn enhance the performance of the CGC operator to be discussed next.

### 3.2. Common Genes Compression (CGC)

The common genes compression operator of PREGA is responsible for compressing and removing the common genes detected by CGD. As outlined in Algorithm 4, the CGC algorithm will first compress the common genes detected by CGD—by choosing a representative for and saving away the information associated with all or each segment of the common genes depending on the applications—and then remove the common genes compressed so that later generations of the GA will only see the chosen representatives. A less number of genes are used to represent the common genes each of which represents a segment of the common genes. For instance, using TSP as an example and assuming that the common genes detected *c*_3_, *c*_4_, and *c*_5_ form a segment of the path, then these genes—and the information associated with them such as the segment of the path they form as well as the length and direction of the segment—can be compressed, that is, represented by a single composite gene, say, *c*_3_′. Once this is done, GA will see only the gene *c*_3_′ at later generations during its convergence process. In other words, each detected segment of the path can be represented by a single composite gene, which is independent of the number of cities of which each segment of the path is composed. Moreover, all the composite genes can be compressed again as the other “noncomposite” genes. It is worth mentioning that we have to take into consideration the relationships between subsolutions to see if they are dependent or independent before they are compressed. If they are independent, all the common genes can be compressed into a single gene. Otherwise, how they are compressed depends on the problem in question and the way the solutions are encoded.

### 3.3. An Example

**Figure 2:**
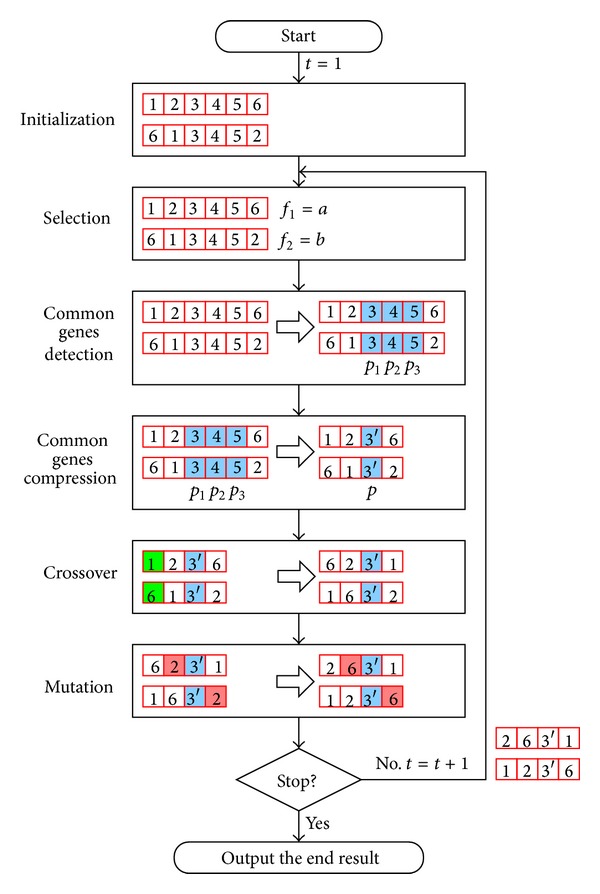
A simple example illustrating how PREGA works. See the text for more detailed explanation.

In this section, we present a simple example to illustrate exactly how PREGA works for the TSP. As Figure 2 shows, the very first step of PREGA is exactly the same as that of GA and is to randomly generate a population of chromosomes. For the purpose of illustration, a population of two chromosomes is generated in this case, and each gene is randomly assigned a distinct city number. Then, the selection operator is applied to select the “good” chromosomes in terms of the fitness value *f*_*i*_ of each chromosome. Then, the CGD and CGC operators, as described in Section 3, are applied for the detection and compression of the genes.

As Figure 2 shows, PREGA differs from GA by adding the CGD and CGC operators as described in Section 3 to eliminate the redundant computations encountered by GA. By doing this, the performance of GA can be significantly enhanced. The example given in Figure 2 shows that the common genes indicated by *p*_1_, *p*_2_, and *p*_3_ are first detected by the CGD operator of PREGA and then compressed by the CGC operator of PREGA, which is denoted by *p*. In other words, after compression, we can choose either one of the three common genes 3, 4, and 5 as the representative to indicate the segment compressed. In this case, we choose 3. To avoid confusion, we use 3′ instead of 3 in Figure 2. After that, the crossover and mutation operators as well as the evaluation of the fitness function will treat each compressed segment as a single pattern until the terminal condition is met. Note that if the genes detected are consecutive, they will be compressed into a single gene. Otherwise, they will be compressed into as few genes as needed; that is, they will be compressed segment by segment.

## 4. Performance Evaluation

In this section, we evaluate the performance of the proposed algorithm by using it to solve the traveling salesman problem. The empirical analysis was conducted on an IBM X3400 machine with 2.0 GHz Xeon CPU and 8 GB of memory using CentOS 5.0 running Linux 2.6.18. All the programs are written in C++ and compiled using g++ (GNU C++ compiler). The benchmarks for the TSP are shown in Table 1. Unless stated otherwise, all the simulations are carried out for 30 runs, with the population size fixed at 80, the crossover probability at 0.5, the per-gene mutation probability at 0.01, the number of generations at 100, and the tournament size at 3 (i.e., 1 out of 3). For all the simulations, PR is started at the second generation.

To improve the quality of the end results of GA, PR, and other evolutionary algorithms, we use several useful technologies to solve the TSP. The nearest-neighbor method [39] is used in creating the initial solution for all the algorithms involved in the simulation. The 2-opt mutation operator [40] is employed as the local search method for fine-tuning the quality of the end results. Unless stated otherwise, all the simulations use HX as the crossover operator by default.

To simplify the discussion of the simulation results of TSP in Tables 2, 3, and 4, we will use the following conventions. Let TGA (traditional GA) [41], HeSEA (heterogeneous selection evolutionary algorithm) [42], SA (simulated annealing) [10], UMDA (univariate marginal distribution algorithm) [43], EHBSA (edge histogram based sampling) [44], ACS (ant colony system) [45], DPSO (discrete particle swarm optimization) [46], and PREGA denote algorithms involved in the simulation. Let *β* ∈ {*D*, *T*} denote either the traveling distance (*β* = *D*) or the computation time (*β* = *T*). Let Δ_*β*_ denote the enhancement of *β*_*ϕ*_ with respect to *β*_*ψ*_ in percentage. Δ_*β*_ is defined as follows:

(3) $$\begin{array}{c} \Delta_{\beta }=\frac{\beta_{\phi }-\beta_{\psi }}{\beta_{\psi }}\times 100\%, \end{array}$$

where *β* is either *D* or *T* for the TSP, and the subscripts *ϕ* and *ψ* are defined as follows.

- For Table 2, *ϕ* = PREGA(*x*) implies *ψ* = TGA(*x*), where *x* denotes the crossover operators [47–49] in use and is either partially matched crossover (PMX), order crossover (OX), heuristic crossover (HX), or edge-recombination crossover (ERX).
- For Tables 3 and 4, *ψ* = TGA, HeSEA, SA, UMDA, EHBSA, ACS, or DPSO, and *ϕ* = PRE*ψ*. Note that in Table 3, to simplify the description, we use PRETGA to indicate PREGA.

Note that for *β* ∈ {*D*, *T*}, the more negative the value of Δ_*β*_, the greater the enhancement.

### 4.1. Impact of Different Removal Strategies

To better understand the impact of the removal bound on the performance of PREGA, we tested several removal bounds—from 0% to 100% with an increment of 10%. 100% means that PREGA may reduce all the genes of chromosomes in the convergence process, whereas 0% means that no genes will be removed; and thus PREGA falls back to GA. More precisely, to simplify the implementation, what we have done is that, after step 2 but before step 3 as shown in Algorithm 2, we check to see if the removal bound is exceeded. If it is exceeded, then steps 3 and 4 will be bypassed. Otherwise, all the common genes detected at step 3 will be removed at step 4 even if it will exceed the removal bound. In other words, we may end up removing a few more genes than the removal bound says.

The experimental results showed that setting the removal bound to 0% (GA) or 100% is better than the others. Although setting the removal bound to 10%, 20%, and up to 90% can also reduce the computation time, setting the removal bound to 100% seems to give a good balance between the computation time and the quality of the end results. It shows that PREGA using 100% removal bound can obtain the best results compared to the other removal bound settings, that is, 10%, 20%, and up to 90%.

A very interesting result to be paid particular attention is that the end result of PREGA using 100% removal bound is better than the others. This result shows that the quality of the PREGA is not linearly proportional to the removal bound. The main reason for this phenomenon is that the local search has to be split into two parts: one is for the common genes and the other is for the noncommon genes. This is required because the common genes have been compressed and thus cannot be mixed up with the noncommon genes. Otherwise, the common genes will become noncommon genes. This situation eventually affects the ability of the local search methods. In other words, with 100% and 0% removal bounds, the search ability of the local search methods is maximized because either all of the genes are either common or noncommon. In the case of 10%, 20%, and up to 90%, however, all the chromosomes are composed of two parts, thus limiting the local search methods to find better subsolutions in a smaller search space instead of the whole search space. This will degrade the quality of the end results, causing the quality of the end results of PREGA to be not linearly proportional to the removal bound.

### 4.2. Impact of Different Kind of Crossover Operators

There are several different crossover operators [48, 49] for the TSP, such as PMX, OX, ERX, and HX. PMX is the most popular and simplest crossover operator, but it lacks searching direction. More recently, many researchers have focused their attention on finding and keeping the building blocks to enhance the performance of GA by either modifying or replacing the operators of GA. In [50], Ruiz et al. designed new crossover operators to identify and maintain the building blocks. In this paper, we use the PMX, OX, HX, and ERX operators to examine the search ability of PREGA when different crossover methods are used. In addition, we have also tested the crossover operators SBOX, SJOX, SB2OX, and SJ2OX [50] to better understand the performance of PREGA with other efficient crossover operators that are designed to avoid disrupting the building blocks on the convergence process. Note that, for the TSP in this paper, we use the* 2-opt mutation* method for reversing two segments (the size of which must be the same) of a tour encoded in a chromosome. For each segment, the edges to the left and right of that segment (if we consider a chromosome as a ring, then the last gene will be next to the first gene or vice versa, and thus there is always a gene to the left or right of a segment) will be replaced by two new edges.

As Table 2 shows, for the TSP, PREGA can effectively reduce the computation time from 80% up to 93.8% using PMX, from 81.82% up to 96.43% using OX, from 87.50% up to 95.91% using HX, from 85.71% up to 95.93% using ERX, from 92.10% up to 94.78% using SBOX, from 91.24% up to 94.57% using SJOX, from 90.78% up to 94.67% using SB2OX, and from 90.17% up to 94.54% using SJ2OX compared to those of traditional GA and GA-based algorithms alone. The simulation results further show that not only does PREGA preserve the accuracy rate of the end results, but also it can even give solutions that are better than those found by the traditional GA and GA-based algorithms alone.

The amount of time that can be reduced and the end results that can be improved depend, to a large extent, on the size of the problem. Our simulation results indicate that the larger the problem, the better the performance of the proposed algorithm. Table 2 also shows that PREGA can even improve the performance of most of the crossover operators, including the crossover operator as complex as HX. This can be easily justified by the following observation. The more complex the crossover operators, the more the computation time required per gene. If the chromosome length or the number of genes can be reduced, it will in turn save the overall computation time. The results in Table 2 show that PREGA is robust even when combining with other efficient crossover operators (e.g., SBOX and SJOX) that use a different method to perform the crossover. Our experimental results also showed that if the original GA or GA-based algorithms do not give a solution that is close to the optimal, PREGA will help arrive at better solution. For example, for the benchmark u2152 using the HX crossover operator, the final result is 73,339.08, which is worse than those using the other crossover operators. PREGA(HX) can, however, save most of the computation time and even improve the quality of the end result by about 2.46%, compared to the others.

### 4.3. Comparison with Evolutionary-Based Algorithms

Finally, for completeness, we compare the performance of traditional GA [41], HeSEA [42], LEM [30], SA [10], UMDA [43], EHBSA [44], ACS [45], and DPSO [46] by applying PR to all of them. Tables 3 and 4 show that not only can PR vastly reduce the computation time of these algorithms, especially for very large data sets, but it can also greatly reduce the computation time of evolutionary-based algorithms that each iteration of which takes a great deal of computation time. Note that the cunning length of EHBSA is 1/3. The inertial weight *ω* of DPSO is 0.5, and the random numbers for determining the influence of personal best and global best *rc*_1_ and *rc*_2_ are, respectively, 0.3 and 0.7. For ACS, the settings are based on those specified in [45]. That is, the population size is 25; the importance of exploitation versus exploration *q*_0_ is 0.9; the importance of pheromone *β* is 2.0; *ρ* is 0.1; and the number of generations is 320.

The results in Table 3 show that because HeSEA takes more computation time than GA per generation, the computation time saved for HeSEA is more than for GA. For instance, the simulation results of the largest benchmark usa13509 show that using GA, the computation time is reduced by a factor of 12.77, whereas using HeSEA, the computation time is reduced by a factor of 22.07. In addition, the results of SA and PRESA highlight a different concept of removing redundant patterns. Because SA is a single-solution-based iterative algorithm, the procedures CGD and CGC have to be modified accordingly. A very simple approach is to remove patterns that are not changed for, say, 1,000 iterations in succession. Furthermore, the simulations of SA and PRESA are carried out for 30 runs, with the initial temperature 1.0 and the change probability *P*(Δ*E*) = exp⁡(−Δ*E*/*k*_*b*_ *T*), where *T* is the temperature and *k*_*b*_ is Boltzmann's constant [10]. The results of Table 3 show that the more the number of solutions (i.e., the larger the population size of the population-based approach is) is used in an iteration, the better the end results is and the longer the computation time is. The results of Table 3 further show that the pattern reduction method can be applied to not only the population-based but also the single-solution-based algorithms where the former finds the common subsolutions to be removed by spatial distribution while the latter finds the common subsolutions to be removed by frequency.

The results in Table 4 show that not only can the proposed algorithm reduce a great deal of the computation time of other efficient evolutionary algorithms such as UMDA and EHBSA, but it can also reduce the computation time of swarm intelligence algorithms such as ACS and DPSO while limiting the loss of the quality of the end result. In other words, the results show that PR can cut down the computation time of evolutionary algorithms, which are themselves either faster or able to provide better results than GA. For instance, even though UMDA, EHBSA, and DPSO are faster than GA by about 44.45%, 29.94%, and 56.35%, respectively, for usa13509, PR can further reduce the computation time of UMDA from 58.61% up to 81.97%, the computation time of EHBSA from 67.46% up to 79.02%, and the computation time of DPSO from 75.00% up to 91.16%. The experimental results show that the proposed algorithm can be used to speed up the performance of all the abovementioned efficient algorithms.

## 5. Analysis of PREGA 

### 5.1. Diversity Analysis

Two of the most important issues in using the pattern reduction method for enhancing the performance of GA or GA-based algorithms are how to ensure the pattern reduction method can effectively reduce the computation time and how to maintain the diversity of the population, that is, the quality of the end results. In this paper, we will discuss the impact of the pattern reduction method on the performance of GA or GA-based algorithms based on three different measures: (1) the average number of genes compressed, (2) the average quality of the end results, and (3) the average size of the search space. In other words, these measures provide an indication of the search ability and the speed of convergence of PREGA.

**Figure 3:**
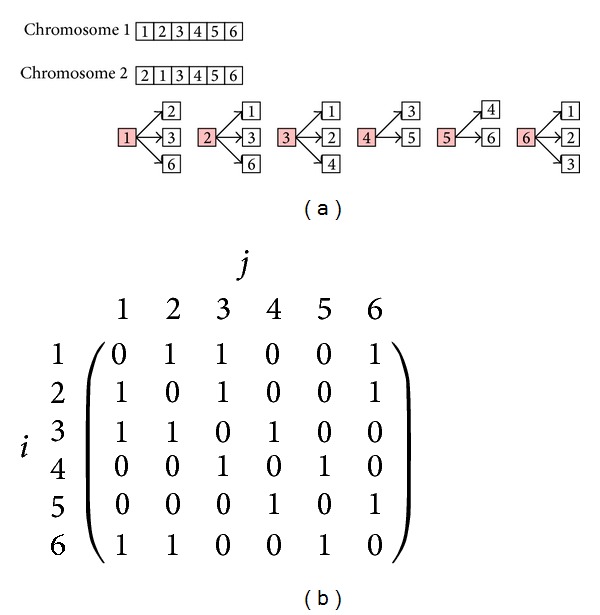
(a) Graphs showing tours encoded in two chromosomes and the outdegree of each city. For instance, the outdegree of city 1 is *d*_1_ = 3. (b) Same information given in (a) represented as an adjacency matrix which makes it easier to understand how the average size of the search space is computed. The number 2 in the denominator in (4) indicates that the adjacency matrix is symmetric in this case.

The search space or diversity of solutions can help us understand whether or not an algorithm is capable of avoiding falling into a local minimum at early generations in the evolution process. In this paper, we use the outdegree of cities as shown in Figure 3 to indicate the search ability of an algorithm. In other words, the higher the outdegree of a city, the higher the search ability. Now, by assuming that the cities next to each other are represented as an adjacency matrix as given in Figure 3(b), the average size of the search space at generation *t*, denoted by ${\overset{-}{S}}^{t}$, is defined as

(4) $$\begin{array}{c} {\overset{-}{S}}^{t}=\frac{1}{2n}\overset{n}{\underset{i=1}{\sum }}\overset{n}{\underset{j=1}{\sum }}e_{i,j}^{t}, \end{array}$$

where *n* is the number of genes (cities) left in each chromosome and *e*_*i*,*j*_^*t*^ = 1 if there exists an edge between cities *i* and *j*; otherwise, *e*_*i*,*j*_^*t*^ = 0. That is, (4) represents the average of the outgoing paths of all the cities currently encoded in all the chromosomes (i.e., not removed). For instance, as Figure 3 shows, sixteen edges exist in all the chromosomes encoding six cities of TSP. The average size of the search space can be computed as (1/(2 × 6)) × 16 = 1.33. This number can help us measure the diversity of the search space of a genetic algorithm at a particular generation.

**Figure 4:**
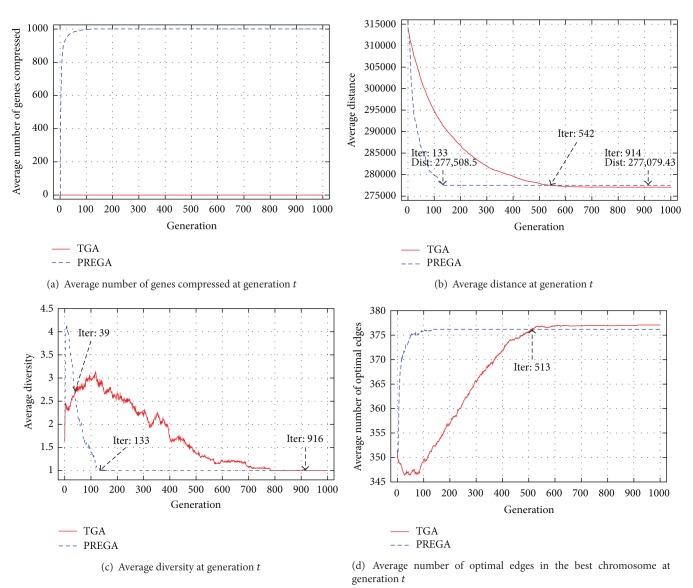
Example illustrating the performance of GA and PREGA for solving the benchmark pr1002.

Figure 4 compares the performance of GA and PREGA for solving the TSP using the simulation result of the benchmark pr1002 as an example.  Figure 4(a) indicates that PREGA can find and remove more common edges than GA, and Figure 4(b) shows that PREGA can find better solutions than GA before generation 542.  Figure 4(c) shows that PREGA can maintain more diversities than the others in the early generations during its evolution process. These results convey a very important message. That is, PREGA would find higher quality result with higher diversities (search space) at the early generations during the convergence process. At the later generations of PREGA, the diversity will become small because it is converging to a stable solution or the global optimum, but our simulation results show that even in this case, PREGA can still reduce most of the redundant computations.

For instance, as Figure 4(c) shows that at about generation 39, the curves of the average diversities of GA and PREGA cross over. That is, the search diversity of PREGA becomes smaller than that of GA at about generation 39, and the gap between these two methods is widened as the number of generations increases. It seems that the search ability of PREGA becomes worse than that of GA. But the result of Figure 4(b) shows that the search ability of PREGA does not eventually decrease between generations 39 and 542. More precisely, in terms of the distance, PREGA finds the solution 277,508.5 at generation 133, even though PREGA is unable to arrive at a better solution afterwards. GA, however, requires about 542 generations to arrive at the same solution 277,508.5 as PREGA. In addition, the final result found by GA is 277,079.43 at generation 914. Then, GA has a very small probability to find a better solution because the search diversity tends to be 1 at generation 916. Now, the most important question is if most of the genes are compressed by PREGA at generation 133 (Figure 4(a)) or later, then will it prevent PREGA from finding better solutions at later generations. According to our simulation results, if either the population size or the problem size is increased, then not all the genes will be removed at the early generations, so the problem will not exist, and PREGA will still outperform GA. Figure 4(b) also indicates that the quality of the final results using GA and PREGA differs by no more than 0.15% (((277,508.5 − 277,079.43)/277,079.43) × 100 = 0.15).

Figure 4(d) gives another measure [42] that can help us understand the performance of GA. The number of genes that is optimal gives us a hint in understanding how fast the optimal solution can be reached by an algorithm. For instance, let us suppose that the path Ψ = {Ψ_1_, Ψ_2_,…, Ψ_*n*_}, where Ψ_*i*_ is the optimal subsolution of an optimal solution for TSP. Now, by assuming that each chromosome is represented as a ring and letting *j* = (*i* + 1)mod⁡*n*, the rate of edges that is optimal in the best chromosome at generation *t*, denoted by *O* ^*t*^, is defined as

(5) $$\begin{array}{c} O^{t}=\overset{n}{\underset{i=1}{\sum }}o_{i,j}^{t}, \end{array}$$

where *n* is the number of genes (cities), and *o*_*i*,*j*_^*t*^ = 1 if there exists an edge that is the optimal subsolution between the pair of genes *i* and *j*; otherwise, *o*_*i*,*j*_^*t*^ = 0. Figure 4(d) shows the probability of edges that are optimal and may end up being in the final solution using GA and PREGA. As indicated in Figure 4(d), PREGA has higher probabilities to find the optimal subsolutions than GA. Also indicated in Figure 4(d) is that even though the average diversity of GA and PREGA crosses over at about generation 513, the final results of GA and PREGA are very similar. More precisely, the difference is about 0.86 optimal edges with a problem of size 1,002.

In summary, the down side of PREGA is that it may quickly converge to a suboptimal solution, but the up side is that the quality of the end result is very close to that of GA. For both GA and PREGA, the number of generations required for the diversity to converge to 1 is in general unpredictable. Using the benchmark pr1002 as an example, if the number of generations performed is 100, PREGA can not only reduce the computation time by about 94.84%, but it can also even enhance the quality of the end result by about 5.08%. However, the average diversities of GA and PREGA at generation 100 are both greater than 1, which indicates that if we let them run longer, they may be able to find a solution that is better than the current one. More precisely, as Figure 4(c) indicates, the average diversity of GA is about 1.4, and the average diversity of PREGA is about 3. Thus, to see what might happen to both GA and PREGA when the diversity approaches 1, the pr1002 benchmark is carried out again for 30 runs and 1,000 generations each run. In average, PREGA takes 0.86 s per run, and GA takes 102.54 s per run. PREGA reduces the computation time by about 99.16% (((0.86 − 102.54)/102.54) × 100 = −99.16) or by a factor of 119.23 compared to GA, and the quality of the end results is very close to each other. In other words, for a large problem, the number of generations required by GA to converge to even a suboptimal solution could be large and is unpredictable. On the other hand, PREGA can quickly provide a solution the quality of which is very close to that of GA even if the size of the problem is large.

### 5.2. Time Complexity of PREGA

The time complexity of genetic algorithm is a very important issue, and it has attracted much attention of many researches [51–53]. In [51], Ambati et al. used information exchange probability, reproduction time, and fitness computation time for estimating the time complexity of GA. According to the results of [51], Ambati et al. presented a GA-based algorithm for solving the TSP, the expected running time of which is *O*(*n*log⁡*n*), where *n* is the number of cities. This is due to the fact that their simulations indicate that “good” solutions can be obtained by GA in *O*(log⁡*n*) generations, even if the size of the TSP is large. In another research [53], Tseng and Yang showed that the time complexity of GA is *O*(*l* *mn* ^2^) for data clustering problem, where *l* is the number of generations, *m* the population size, and *n* the number of patterns.

In this paper, we* assume* that the time complexity of the traditional genetic algorithm is (*nml*), where *n* is the number of genes, *m* the number of chromosomes, and *l* the number of generations. This can be easily justified by the following analysis on the time complexity of the fitness function, selection, crossover, and mutation operators used by the traditional genetic algorithm as far as certain conditions are met. For instance, suppose that tournament selection is used as the selection operator, and its size is *k* (a constant that is far less than *m*). Let us further suppose that one point crossover with probability *p*_*c*_ and one point mutation with each gene having probability *p*_*m*_ which are mutated are used where *p*_*c*_ and *p*_*m*_ are less than 1. The selection operator takes *km* time at each iteration, because GA needs to randomly select *k* chromosomes from a set of *m* chromosomes to find the best one and performs this procedure *m* times. The one point crossover will exchange the information about *p*_*c*_ *mn* time, and the mutation operator will take about *p*_*m*_ *mn* time, and the fitness function takes *mn* time. The overall complexity of the traditional genetic algorithm is thus *O*(*nml*) (e.g., *k*, *p*_*c*_, and *p*_*m*_ are parameters (constants) that you choose before a simulation is carried out, and all the simulation results given in Section 4 have *k* = 3 (≪*m* = 80), *p*_*c*_ = 0.5 (<1), and *p*_*m*_ = 0.01 (≪1)) where *m* and *n* are as defined above, *l* is the number of generations required to converge, and the assumption that all the operators do not take more than *n* or *m* time holds. Otherwise, the time complexity could be *O*(*n* ^2^ *ml*) or *O*(*nm* ^2^ *l*). In the ideal case, the pattern reduction algorithm can reduce the time complexity of GA from *O*(*nml*) to *O*(*nm*). This can be easily proved by letting Δ (0 < Δ < 1) be a constant indicating the percentage of patterns retained at each iteration. In other words, 1 − Δ is the percentage of genes removed at each iteration in all chromosomes. Then,

(6) ∑i=0l−1Δinm=nm∑i=0l−1Δi≤nm∑i=0∞Δi=nm11−Δ=O(nm).

In summary, the time complexity of PREGA is bound from above by *O*(*nml*) and from below by *O*(*nm*). In the best case, when the PREGA algorithm is started at the very first iteration and the removal bound is set to 100%, the time complexity will be *O*(*nm*). In the worst case, if PREGA cannot detect any common genes to be removed, then PREGA will fall back to GA, and the time complexity will be *O*(*nml*). In other words, the time complexity of PREGA depends on (1) the iteration at which PREGA starts, (2) the number of patterns removed at each iteration, and (3) the removal bound, which is defined to be “up to *x*% of the genes detected can be removed,” though in practice, a little bit more than the removal bound of genes can be removed to simplify the implementation (more details can be found in Section 4.1). Our simulation results showed that PREGA can reduce the computation time of GA from about 80% to 95.32% when the removal bound is set to 100% for complex data sets. The results further showed that if the number of generations of GA is set to an even larger value, we can reduce the time complexity of GA to approach that of the ideal case, that is, *O*(*nm*).

## 6. Conclusion

This paper presents a novel technique for reducing the computation time of GA or GA-based algorithms based on the notion of pattern reduction. To evaluate the performance of the proposed algorithm, we use it to solve the traveling salesman problem, the benchmarks of which range in size from 130 to 13,509 cities. All our simulation results showed that the proposed algorithm can effectively cut down the computation time of GA and its variants, especially in cases where the data sets are large. Our simulation results further showed that the proposed algorithm can significantly reduce the computation time of the state-of-the-art heuristic algorithms we compared in the paper, such as ACO and PSO, even though these algorithms themselves are very efficient in solving the combinatorial optimization problems. In the future, our focus will be on enhancing the performance of the proposed algorithm and widening the domains of its application.

## Figures and Tables

**Algorithm 1:**
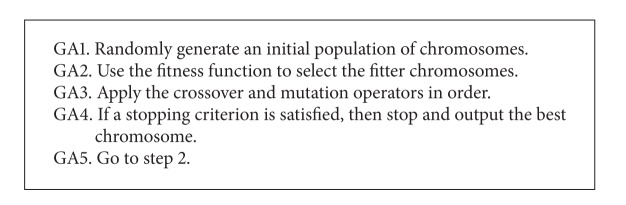
Outline of traditional genetic algorithm (GA).

**Algorithm 2:**
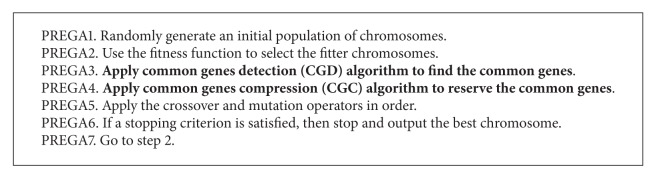
Outline of pattern reduction enhanced genetic algorithm (PREGA).

**Algorithm 3:**
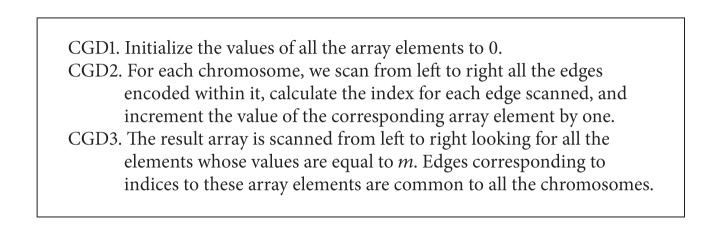
Outline of common genes detection algorithm.

**Algorithm 4:**
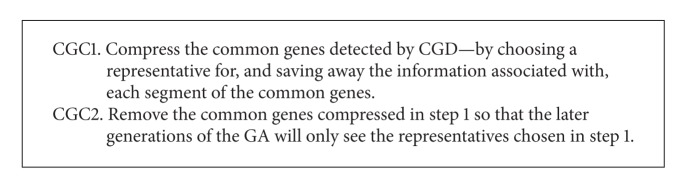
Outline of common genes compression algorithm.

**Table 1:** Data sets for TSP.

Data set	Number of cities	Optimum
ch130	130	6,110
ch150	150	6,528
d198	198	15,780
a280	280	2,579
pcb442	442	50,778
d493	493	35,002
u574	574	36,905
u724	724	41,910
pr1002	1,002	259,045
u1060	1,060	224,094
d1291	1,291	50,801
u1432	1,432	152,970
d1655	1,655	62,128
u2152	2,152	64,253
pr2392	2,392	378,032
pcb3038	3,038	137,694
fnl4461	4,461	182,566
usa13509	13,509	19,982,889

**Table 2:** Simulation results of using different crossover operators.

Data set	PREGA(PMX)			PREGA(OX)			PREGA(HX)			PREGA(ERX)		
	Δ_*D*_	*c*_*v*_	Δ_*T*_	Δ_*D*_	*c*_*v*_	Δ_*T*_	Δ_*D*_	*c*_*v*_	Δ_*T*_	Δ_*D*_	*c*_*v*_	Δ_*T*_
ch130	0.92	(1.49%)	−80.00	0.85	(1.25%)	−81.82	1.56	(1.51%)	−87.50	1.95	(1.50%)	−85.71
ch150	1.67	(0.62%)	−84.62	1.53	(0.69%)	−85.71	1.43	(0.59%)	−90.00	1.31	(0.51%)	−88.24
d198	0.80	(1.05%)	−86.36	0.44	(1.46%)	−86.96	0.42	(0.90%)	−90.91	0.60	(0.81%)	−89.29
a280	0.39	(1.73%)	−86.36	0.09	(1.63%)	−91.11	−0.71	(1.68%)	−91.80	0.50	(1.37%)	−90.38
pcb442	1.18	(0.66%)	−90.18	0.55	(0.72%)	−92.98	−2.59	(0.69%)	−93.75	1.96	(0.99%)	−91.2
d493	2.74	(4.39%)	−81.76	−1.15	(1.04%)	−92.62	−2.67	(2.00%)	−92.55	1.16	(1.10%)	−90.18
u574	−1.05	(0.80%)	−91.17	−2.23	(0.59%)	−95.10	−5.51	(0.84%)	−94.66	0.95	(0.69%)	−93.38
u724	−0.78	(0.75%)	−87.41	−2.26	(0.64%)	−95.02	−5.11	(2.39%)	−94.58	1.22	(0.70%)	−93.16
pr1002	−1.48	(0.58%)	−91.94	−2.38	(0.65%)	−95.77	−5.08	(2.79%)	−94.84	0.81	(0.79%)	−94.79
u1060	−1.14	(0.99%)	−89.69	−2.87	(0.63%)	−95.52	−3.69	(4.31%)	−94.55	1.18	(0.98%)	−94.10
d1291	0.17	(0.74%)	−93.80	−0.10	(0.95%)	−96.43	−3.38	(0.86%)	−95.91	0.18	(1.04%)	−95.93
u1432	3.53	(4.36%)	−80.50	−3.68	(0.50%)	−95.19	−2.76	(4.20%)	−93.74	1.07	(1.00%)	−93.36
d1655	−0.85	(1.75%)	−91.79	−2.06	(1.10%)	−96.32	−3.39	(2.57%)	−95.47	0.98	(0.92%)	−95.36
u2152	−0.77	(2.67%)	−89.53	−2.05	(0.52%)	−96.38	−3.00	(2.41%)	−95.71	0.78	(0.52%)	−95.69

Average	0.38		−87.51	−1.09		−92.64	−2.46		−93.28	1.05		−92.2

Data set	PREGA(SBOX)			PREGA(SJOX)			PREGA(SB2OX)			PREGA(SJ2OX)		
	Δ_*D*_	*c*_*v*_	Δ_*T*_	Δ_*D*_	*c*_*v*_	Δ_*T*_	Δ_*D*_	*c*_*v*_	Δ_*T*_	Δ_*D*_	*c*_*v*_	Δ_*T*_

ch130	1.03	(1.51%)	−92.65	0.93	(1.54%)	−91.24	1.02	(1.45%)	−91.11	1.37	(1.71%)	−90.33
ch150	1.35	(0.58%)	−92.60	1.58	(0.56%)	−92.33	1.57	(0.55%)	−92.66	1.41	(0.56%)	−92.48
d198	0.55	(1.00%)	−92.36	0.85	(0.92%)	−91.81	0.67	(0.85%)	−90.78	0.86	(0.82%)	−90.17
a280	0.84	(1.25%)	−92.10	1.55	(1.58%)	−93.13	−0.11	(1.67%)	−91.58	0.68	(1.50%)	−91.98
pcb442	1.81	(0.94%)	−93.48	1.50	(0.80%)	−93.02	0.95	(0.89%)	−93.02	0.80	(1.03%)	−93.21
d493	1.07	(0.97%)	−92.63	0.85	(1.07%)	−92.98	0.16	(0.75%)	−91.61	0.63	(0.82%)	−90.77
u574	−0.13	(0.85%)	−92.52	0.06	(0.90%)	−93.07	−1.17	(0.64%)	−92.28	−1.31	(0.80%)	−92.45
u724	0.75	(0.79%)	−93.78	0.11	(0.57%)	−93.42	−0.85	(0.71%)	−93.70	−1.00	(0.73%)	−93.39
pr1002	−0.17	(0.50%)	−94.06	−0.26	(0.64%)	−93.64	−1.65	(0.69%)	−94.17	−1.76	(0.74%)	−93.85
u1060	−0.09	(0.63%)	−93.35	0.09	(0.72%)	−94.13	−1.39	(0.51%)	−93.11	−2.05	(0.93%)	−92.00
d1291	0.55	(1.08%)	−93.49	0.48	(0.92%)	−92.68	−0.22	(0.79%)	−92.78	−0.19	(0.82%)	−92.69
u1432	−0.20	(0.74%)	−93.41	−0.11	(0.73%)	−93.13	−2.08	(0.48%)	−93.48	−2.18	(0.77%)	−93.49
d1655	−0.22	(0.62%)	−94.08	−0.55	(0.65%)	−94.13	−1.60	(0.86%)	−94.29	−1.02	(0.91%)	−94.2
u2152	−0.18	(0.55%)	−94.78	−0.26	(0.57%)	−94.57	−0.38	(0.48%)	−94.67	−0.66	(0.52%)	−94.54

Average	0.50		−93.23	0.49		−93.09	−0.36		−92.80	−0.32		−92.54

*T*: time in seconds; *c*_*v*_: coefficient of variation, which is defined to be *c*_*v*_ = *σ*/*μ*, where *μ* is either *D* or Δ_*D*_.

**Table 3:** Simulation results of GA, HeSEA, LEM, and SA.

Data set	PREGA			PREHeSEA			PRELEM			PRESA		
	Δ_*D*_	*c*_*v*_	Δ_*T*_	Δ_*D*_	*c*_*v*_	Δ_*T*_	Δ_*D*_	*c*_*v*_	Δ_*T*_	Δ_*D*_	*c*_*v*_	Δ_*T*_
a280 (*c*_*v*_)	−0.71	(1.68%)	−91.84	0.22	(1.47%)	−94.79	1.17	(1.90%)	−95.92	−0.07	(1.48%)	−57.62
*b*_30_	2,679.44			2,681.44			2,671.60			2,743.77		
u574 (*c*_*v*_)	−5.51	(0.84%)	−94.76	−5.88	(0.65%)	−93.14	1.25	(0.83%)	−95.92	0.02	(1.17%)	−52.54
*b*_30_	38,837.70			39,211.40			39,156.40			39,448.30		
u724 (*c*_*v*_)	−5.12	(2.39%)	−94.61	−3.95	(0.77%)	−93.75	1.04	(0.89%)	−96.14	−0.27	(0.95%)	−52.68
*b*_30_	44,399.10			44,347.70			44,388.70			45,044.90		
u1060 (*c*_*v*_)	−3.69	(4.31%)	−94.55	−5.52	(2.28%)	−93.97	0.85	(0.77%)	−96.25	−0.29	(1.01%)	−49.33
*b*_30_	241,758.00			238,428.00			237,896.00			238,928.00		
u1432 (*c*_*v*_)	−2.76	(4.20%)	−93.76	−0.45	(3.80%)	−93.78	1.27	(0.80%)	−95.97	0.13	(0.75%)	−53.26
*b*_30_	164,093.00			165,570.00			162,950.00			162,973.00		
pr2392 (*c*_*v*_)	−3.32	(3.34%)	−95.06	−5.58	(2.43%)	−95.89	0.14	(0.46%)	−96.61	−0.25	(0.58%)	−53.20
*b*_30_	405,470.00			405,612.00			407,597.00			419,796.00		
pcb3038 (*c*_*v*_)	−0.55	(4.64%)	−94.72	−0.97	(4.22%)	−95.34	0.40	(0.66%)	−95.73	−0.05	(0.73%)	−59.18
*b*_30_	148,567.00			148,293.00			148,353.00			152,449.00		
fnl4461 (*c*_*v*_)	−4.09	(4.52%)	−95.32	−1.02	(3.06%)	−95.58	0.00	(0.51%)	−95.19	0.12	(0.36%)	−61.21
*b*_30_	195,074.00			195,938.00			195,907.00			202,525.00		
usa13509 (*c*_*v*_)	−0.77	(5.23%)	−92.18	6.09	(0.42%)	−95.47	−0.17	(0.28%)	−94.89	−1.41	(0.46%)	−85.03
*b*_30_	21,500,000.00			23,700,000.00			21,900,000.00			22,600,000.00		

Average	−2.95		−94.09	−1.90		−94.63	0.66		−95.85	−0.23		−58.23

*T*: time in seconds; *b*_30_: best solution in 30 runs; *c*_*v*_: coefficient of variation as defined in Table 2.

**Table 4:** Simulation results of UMDA, EHBSA, ACS, and DPSO.

Data set	PREUMDA			PREEHBSA			PREACS			PREDPSO		
	Δ_*D*_	*c*_*v*_	Δ_*T*_	Δ_*D*_	*c*_*v*_	Δ_*T*_	Δ_*D*_	*c*_*v*_	Δ_*T*_	Δ_*D*_	*c*_*v*_	Δ_*T*_
a280 (*c*_*v*_)	6.73	(3.14%)	−66.00	1.59	(1.85%)	−69.82	−3.31	(1.24%)	−22.14	−5.13	(2.56%)	−75.00
*b*_30_	2,722.10			2,687.78			2,625.38			2,701.27		
u574 (*c*_*v*_)	−4.52	(0.71%)	−58.61	0.52	(0.80%)	−67.46	0.64	(0.86%)	−69.01	−7.85	(4.25%)	−84.39
*b*_30_	39,013.00			39,392.90			38,299.70			39,031.40		
u724 (*c*_*v*_)	−4.14	(0.67%)	−59.47	0.39	(0.78%)	−67.95	−0.85	(0.77%)	−78.31	−4.20	(4.19%)	−84.66
*b*_30_	44,419.90			44,347.50			42,805.40			44,854.00		
u1060 (*c*_*v*_)	−4.76	(0.70%)	−73.35	−0.09	(0.76%)	−74.11	1.46	(0.66%)	−80.67	−1.35	(3.24%)	−87.93
*b*_30_	239,226.00			238,155.00			234,396.00			242,863.00		
u1432 (*c*_*v*_)	−2.93	(0.65%)	−70.61	0.51	(0.67%)	−73.32	0.29	(0.66%)	−73.85	−0.11	(0.65%)	−76.42
*b*_30_	163,520.00			163,504.00			161,204.00			183,637.00		
pr2392 (*c*_*v*_)	−3.51	(0.46%)	−81.97	−0.90	(0.52%)	−75.59	2.51	(0.79%)	−89.26	−0.68	(0.42%)	−91.16
*b*_30_	406,302.00			404,867.00			401,594.00			452,346.00		
pcb3038 (*c*_*v*_)	−3.36	(0.33%)	−69.17	−0.86	(0.32%)	−76.67	4.58	(0.57%)	−91.07	−0.13	(0.55%)	−86.84
*b*_30_	148,258.00			148,374.00			149,715.00			165,307.00		
fnl4461 (*c*_*v*_)	−3.60	(0.22%)	−73.20	−1.46	(0.29%)	−79.02	2.09	(0.63%)	−92.30	−0.16	(0.38%)	−86.35
*b*_30_	195,063.00			195,747.00			200,487.00			219,180.00		
usa13509 (*c*_*v*_)	−4.14	(0.27%)	−70.52	−2.39	(0.26%)	−73.01	1.16	(0.34%)	−93.45	−0.49	(0.31%)	−88.24
*b*_30_	21,479,200.00			21,531,300.00			22,584,600.00			24,222,100.00		

Average		−2.69	−69.21	0.3		−73.00	0.95		−76.67	−2.23		−84.55

*T*: time in seconds; *b*_30_: best solution in 30 runs; *c*_*v*_: coefficient of variation as defined in Table 2.
